# Insights on the Pathogenesis of Aneurysm through the Study of Hereditary Aortopathies

**DOI:** 10.3390/genes12020183

**Published:** 2021-01-27

**Authors:** Tyler J. Creamer, Emily E. Bramel, Elena Gallo MacFarlane

**Affiliations:** 1 McKusick-Nathans Department of Genetic Medicine, Johns Hopkins University School of Medicine, Baltimore, MD 21205, USA; tcreamer@jhmi.edu (T.J.C.); ebramel1@jhmi.edu (E.E.B.); 2 Department of Surgery, Johns Hopkins University School of Medicine, Baltimore, MD 21205, USA; 3 Predoctoral Training in Human Genetics and Molecular Biology, Johns Hopkins University School of Medicine, Baltimore, MD 21205, USA

**Keywords:** aorta, aortopathy, aneurysm, thoracic aortic aneurysm, Marfan syndrome, Loeys–Dietz syndrome, familial thoracic aortic aneurysm, TGF-β, extracellular matrix

## Abstract

Thoracic aortic aneurysms (TAA) are permanent and localized dilations of the aorta that predispose patients to a life-threatening risk of aortic dissection or rupture. The identification of pathogenic variants that cause hereditary forms of TAA has delineated fundamental molecular processes required to maintain aortic homeostasis. Vascular smooth muscle cells (VSMCs) elaborate and remodel the extracellular matrix (ECM) in response to mechanical and biochemical cues from their environment. Causal variants for hereditary forms of aneurysm compromise the function of gene products involved in the transmission or interpretation of these signals, initiating processes that eventually lead to degeneration and mechanical failure of the vessel. These include mutations that interfere with transduction of stimuli from the matrix to the actin–myosin cytoskeleton through integrins, and those that impair signaling pathways activated by transforming growth factor-β (TGF-β). In this review, we summarize the features of the healthy aortic wall, the major pathways involved in the modulation of VSMC phenotypes, and the basic molecular functions impaired by TAA-associated mutations. We also discuss how the heterogeneity and balance of adaptive and maladaptive responses to the initial genetic insult might contribute to disease.

## 1. Introduction

Aneurysms are permanent, localized dilatations of an artery greater than 50% of the normal diameter. They progressively dilate while remaining mostly asymptomatic until a life-threatening rupture and/or dissection occurs [1]. Prophylactic surgical repair remains the only proven method to prevent risk of death caused by mechanical failure of the vessel [2]. Aneurysms can develop both in the thoracic and abdominal aorta [3]. Aneurysms that affect the abdominal aorta are more common, tend to occur in older individuals, and have no known monogenic cause, although multiple candidate risk loci have been reported [4,5,6]. While less common, thoracic aortic aneurysms (TAA) can develop in the absence of cardiovascular risk factors, affect younger individuals, and have a higher degree of heritability [6,7]. Although a hereditary predisposition to TAA confers an increased risk of aortopathy to all segments of the vessel, pathogenic mechanisms can differ depending on the specific aortic location [8,9]. For example, dissections of the thoracic descending aorta can occur even when dilation is limited or absent and as a complication of proximal aortic repair [10,11,12]. Hereditary forms of TAA are subdivided into syndromic and non-syndromic depending on the presence or absence of manifestations in other organ systems [13]. Syndromic forms of TAA occur in patients affected by connective tissue disorders such as Marfan syndrome (MFS) and Loeys–Dietz syndrome (LDS) [13], all of which have manifestations in organ systems other than the aorta. In contrast, TAAs in hereditary non-syndromic thoracic aortic disease are not usually associated with overt defects in other connective tissues [13]. Several causative genes for both syndromic and non-syndromic TAA have been identified, leading to a better understanding of the mechanisms by which this condition develops [14]. In this review, we summarize critical features of the healthy aortic wall, the molecular functions compromised by TAA-associated mutations, and the proposed mechanisms by which they disrupt aortic homeostasis. Although similarities exist between all forms of TAA, we primarily focus on the pathogenesis of aneurysm of the proximal portion of aorta. We also discuss how mechanistic insights on TAA pathogenesis, and understanding of the regional heterogeneity of the aorta, might instruct the development of medical therapies.

## 2. Structure and Features of the Normal Aortic Wall

**Figure 1:**
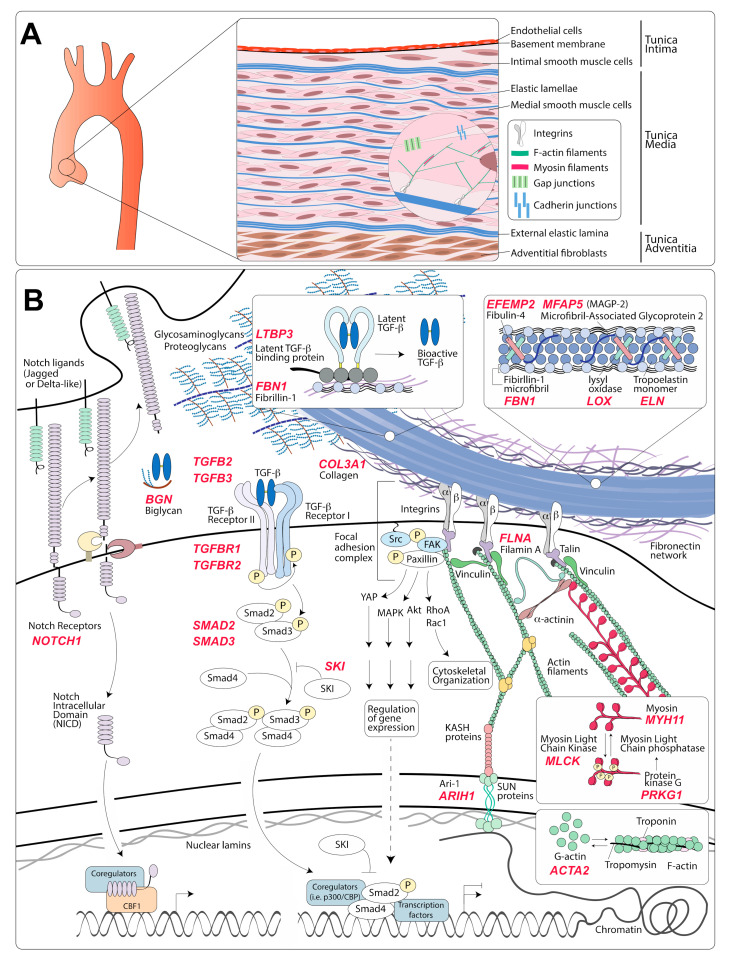
**Organization of the aortic wall and representation of molecular pathways affected in hereditary aneurysm disorders.** (**A**) Schematic representation of the thoracic aorta magnified to illustrate the composition of the three aortic layers, cell types, and intracellular features of vascular smooth muscle cells (VSMCs). The aortic media is composed of alternating layers of VSMCs and elastic lamellae. Integrins link the actin cytoskeleton to elastic fibers and other extracellular matrix components (ECM). Cadherin and gap junctions allow sharing of mechanical and biochemical information between adjacent cells. (**B**) Representation of molecular components involved in the transmission of mechanical and biochemical information in the vascular wall. Causal mutations for hereditary thoracic aortic aneurysm (TAA) have been found in proteins coded by genes noted in red. Elastic fibers are composed of a core of elastin surrounded by microfibrils; although fibrillin-1 is the main component of microfibrils, they also include other glycoproteins such as microfibril-associated glycoproteins and fibulins. Maturation of elastic fibers requires lysyl oxidase enzymatic activity, which catalyzes a crosslinking reaction between monomers of tropoelastin. Mechanical stimuli are transmitted from the elastic fibers to the VSMCs via integrins, which are heterodimeric transmembrane proteins that bind to matrix proteins, including fibrillin-1, via multi-protein complexes called focal adhesions. Focal adhesions contain several adaptor proteins anchoring integrins to the actin and myosin cytoskeleton and to the linker of nucleoskeleton and cytoskeleton (LINC) complex. Myosin light chain kinase (MLCK) promotes contraction by phosphorylation of myosin regulatory light chain (RLC), whereas myosin light chain phosphatase (MLCP) dephosphorylates RLC and promotes relaxation. Integrins transfer mechanical inputs from the cytoskeleton to the ECM and from the ECM to the cytoskeleton. Integrin engagement promotes the polymerization of globular actin (G-actin) into actin filaments (F-actin). Several signaling molecules are regulated by focal adhesions, including focal adhesion kinase (FAK), mitogen-activated protein kinases (MAPK), Akt, Rac and Rho GTPases, and Yes-associated protein (YAP). Transforming growth factor-β (TGF-β) ligands are secreted as inactive latent complexes that are converted into a bioactive TGF-β ligand by chemical, mechanical, and enzymatic processes in the ECM. TGF-β ligand binds to the TGF-β receptor complex and induces the phosphorylation of Smad2 and Smad3. Then, phosphorylated Smad2 and Smad3 (pSmad2/3) can bind to Smad4 and translocate to the nucleus, where they regulate TGF-β responsive genes. Sloan-Kettering Institute (SKI) is a repressor of TGF-β signaling that inhibits the association of pSmad2/3 with Smad4, and with other transcriptional co-regulators such as p300. The binding of Notch to its cognate receptor induces a conformational change that triggers multiple proteolytic processing steps that release the Notch intracellular domain (NICD) from the membrane and promote its translocation to the nucleus, where it regulates transcription via interaction with other factors.

The healthy arterial wall is composed of three distinct layers: the tunica intima, the tunica media, and the tunica adventitia [15] (Figure 1A). The primary cellular component of the tunica intima is endothelial cells, which are arranged in a monolayer directly facing the lumen of the vessel [15]. Endothelial cells are anchored to a basement membrane of collagen, laminin, and proteoglycans and provide a semi-selective, flexible barrier that responds to shear stress, modulates immune responses, and participates in vascular repair [16]. The subendothelial matrix, which in larger animals can include intimal smooth muscle cells, is separated from the next outer layer by the internal elastic lamina [15].

The tunica media is the thickest portion of the arterial wall and the layer that endows the vessel with structural integrity and mechanical functionality [15,17]. It consists of multiple, circumferentially arranged layers of vascular smooth muscle cells (VSMCs) alternating with layers of fenestrated elastic lamellae, which are composed of elastic fibers. VSMCs form extensions that connect to the lamellar layers and are visible by transmission electron micrographs [18,19,20]. In addition, adherens and gap junctions transmit mechanical and biochemical information between adjacent VSMCs [21,22]. This ordered structure is organized within a complex matrix of proteoglycans, glycoproteins, glycosaminoglycans, and various types of collagen [20]. Medial VSMCs are critical to the establishment of the extracellular matrix (ECM) during development, its maintenance during adult life, and its degradation and replacement in response to mechanical and biochemical cues (remodeling) [17,20,23]. 

The external elastic lamina separates the media from the third and outermost layer of the arterial wall, the adventitia, which is comprised of fibroblasts and, according to some studies, progenitor cells with VSMC differentiation potential, all located within a collagen-rich matrix [24,25,26,27]. This outer layer provides mechanical support to the vessel under both physiological and pathological conditions [28,29]. Adventitial fibroblasts serve a role analogous to that of medial VSMCs by establishing and remodeling the ECM of the adventitial layer in response to developmental and mechanical cues [28]. While thinner arteries comprising of few elastic lamellae, such as those of small rodents, are adequately nourished from the lumen, larger arteries require an external access to a blood supply. In these thicker vessels, the adventitial layer hosts a series of small arteries called vasa vasorum (vessels of the vessel), which supply oxygen and nutrients to the outer two-thirds of the vessel wall [30].

## 3. Modulation of Medial VSMC Phenotype

Analysis of aneurysmal tissue obtained from both patients and mouse models reveals common end-point characteristics that are observed regardless of etiology [31,32]. Although both the intimal and adventitial layers participate in the disease process [33,34,35], the progressive mechanical weakening of the vessel is caused by the “degeneration” of the tunica media [31,32]. Features associated with medial degeneration include fragmentation of elastic lamellae, increased and/or abnormal deposition of collagen and proteoglycans, and increased wall permeability [36,37,38,39,40,41]. Although glycosaminoglycan and proteoglycans serve important physiological functions and help elastic lamellae resist hemodynamic forces [42,43], the accumulation of these molecules in TAA, either by increased deposition or decreased degradation, obstructs elastic fibers, increases the swelling pressures on the vessel wall, and has been strongly associated with aortic dissection and rupture in a mouse model of MFS [36,40,42,44,45,46]. In our discussion, we focus on the role of VSMCs in initiating and promoting the processes that culminate in media degeneration and mechanical failure of the vessel; we refer to other recent reviews for analysis of the role of endothelial cells and adventitial fibroblasts in arterial pathology [47,48].

In the healthy adult aorta, VSMCs are quiescent and primarily dedicated to regulation of vascular tone through their contractile function [49]. This phenotype can be modified by environmental cues received via receptors that bind ECM proteins and by receptors that bind to soluble or membrane-bound ligands [49,50,51,52]. During TAA development, the dysfunctional integration of mechanical and biochemical signals promotes VSMC transition from a “contractile” to a “synthetic” phenotype, which is characterized by elevated synthetic and proliferative activity and the downregulation of contractile proteins [49,53,54]. In both syndromic and non-syndromic forms of TAA, aortic VSMCs are characterized by an enlarged endoplasmic reticulum suggestive of high synthetic activity, decreased number of actomyosin filaments, and increased expression and secretion of matrix degrading enzymes [55,56,57,58,59,60,61]. Although these phenotypic changes can in some cases participate in vascular repair, they also lead to the deposition of dysfunctional matrix, given that the ability of adult VSMCs to synthetize and properly organize components of the elastic lamellae is limited [23]. In turn, degradation of the ECM caused by excess proteolytic activity can lead to the activation of signaling pathways that are regulated by ECM proteins and/or by ECM-dependent signaling [53].

Multiple signaling pathways are dysregulated in TAA in consequence of all these processes, including those activated by angiotensin II, insulin-like growth factor-1 (IGF-1), platelet-derived growth factor (PDGF), and TGF-β [62,63,64,65]. Mitochondrial dysfunction and the presence of inflammatory cells have also been reported in TAA, although these may represent late features secondary to advanced pathogenesis [66,67,68,69,70]. All these events participate in progressively decreasing the ability of the aortic media to sustain mechanical stress, leading first to dilation and eventually to dissection and/or rupture [53]. It is important to note, however, that dissection and rupture, although clinically overlapping with TAA, can also occur in the absence of these features [71].

Numerous environmental cues can modulate VMSC phenotypes. Although there is significant cross-regulation, these signals can be broadly classified as dependent on mechanical connections between the ECM and VSMCs, or as being primarily activated by cell receptors that bind soluble or cell-bound ligands. We will briefly summarize the basic components of these modulatory pathways by focusing on those elements that have been found to be directly involved in the pathogenesis of hereditary TAA (Figure 1B).

### 3.1. Modulation of VSMC Phenotype via Interaction with the ECM

VSMCs connect to the ECM via integrins and non-integrin matrix receptor molecules. Non-integrin receptors include CD44 (which binds to hyaluronan, a glycosaminoglycan), discoidin domain receptor tyrosine kinases (which are upregulated in response to injury and bind to collagen), and the receptor for advanced glycation end-products (RAGE), all of which are reviewed in references [21,72].

Integrins, a family of heterodimeric transmembrane receptors formed by the dimerization of an α and a β subunit, are the main transducers of mechanical cues between the ECM and VSMCs. Each heterodimeric combination of integrin molecules binds to specific ECM components [73,74,75]. Upon binding to their corresponding extracellular ligand, integrins undergo conformational changes and localize to focal adhesions, multi-protein complexes that connect ligand-bound integrins to the cytoskeleton through anchoring proteins that bind directly to actin filaments [76,77,78]. The engagement of focal adhesions promotes the activation of several signaling pathways and stretch-dependent polymerization of globular actin (G-actin) into filaments (F-actin) [76,77,78].

Integrin can transduce both biochemical and mechanical information to VSMCs and thus modulate their phenotype in response to the presence of specific ligands in the ECM and in response to shear stress, tension, or “stiffness” [28,74,79]. Integrins α_5_*β*_1_ and α_V_*β*_3_ connect the actin–myosin cytoskeleton to fibrillin-1 present in microfibrils [23,80]. Microfibrils enclose and support the core of the elastic fibers, which is composed of polymerized and crosslinked monomers of tropoelastin [23,80] (Figure 1B). Other ECM proteins associated with elastic fibers include microfibril-associated glycoproteins (MAGPs) and fibulins (reviewed in detail in reference [23]). The structure comprising of elastic fibers, integrin receptors, anchoring proteins, and cytoskeletal contractile filaments is referred to as the “elastin-contractile unit” [18,81,82]. This structure is critical for transduction of mechanical information that modulates VSMCs phenotypes and helps maintain these cells in a quiescent, contractile non-proliferative state [83,84,85,86].

In VSMCs, the actin–myosin unit is composed of antiparallel filaments of a smooth muscle specific form of actin (α-smooth muscle actin, α-SMA) and myosin filaments that include dimers of a smooth muscle specific myosin heavy chain (smooth muscle myosin heavy chain, SM-MHC) [87]. This structure is responsible for VSMC’s contraction and the regulation of blood pressure through vasoconstriction. Contraction is promoted by signals that increase the intracellular calcium ion (Ca^2+^) concentration, either through the opening of ion channels on the plasma membrane or by release from internal stores [88,89,90]. Ca^2+^ binds to and activates calmodulin, which in turn activates myosin light chain kinase (MLCK), the enzyme that phosphorylates myosin light regulatory chains and initiates myosin-dependent conversion of ATP into mechanical energy [88,89,90]. Myosin light chain phosphatase (MLCP) dephosphorylates myosin light regulatory chains and induces relaxation upon decrease of intracellular Ca^2+^ concentration [88,89,90]. The balance between contraction and relaxation is regulated by signaling pathways that control levels of intracellular Ca^2+^ concentration and by those that modulate the sensitivity of the actin–myosin unit to changes in Ca^2+^ concentration via post-translational modifications [88,89,90].

In addition to controlling vascular tone, the contraction of the actin–myosin unit regulates the composition and function of focal adhesions, reinforcing the connections between anchoring proteins, integrins, and ECM, and promoting actin polymerization [91]. Although mechanisms are not fully elucidated, myosin-dependent force generation may affect focal adhesions by inducing tension-dependent conformational changes in both integrins and anchoring proteins, which can then recruit and activate other signaling effectors [92,93,94,95,96]. Accordingly, pharmacological or genetic inactivation of components of the actin–myosin contractile unit has been shown to result in the defective maturation and function of focal adhesions [91,97].

Mechanical stretch can directly activate several cell receptors, kinases, proteases, and other enzymes [50,98,99,100,101,102,103]. Integrin engagement links force generation to modulation of transcriptional activity by the activation of focal adhesion kinase (FAK) and other focal adhesion proteins that shuttle from the cytoplasm to the nucleus, where they regulate the activity of transcription factors such as GATA4 and p53 [104,105,106,107,108,109]. Moreover, force generation via the actomyosin cytoskeleton modulates the regulatory functions of nuclear proteins through the linker of nucleoskeleton and cytoskeleton (LINC) complex [107,110,111,112,113].

### 3.2. Modulation of VSMC Phenotype by Soluble or Membrane-Bound Ligands

Growth factors, cytokines, and membrane-bound ligands bind to cell receptors expressed on VSMCs and activate signaling pathways that act on several transcription factors, regulating proliferation, synthesis of contractile proteins, deposition of new matrix components, and expression of matrix-degrading enzymes [49,54]. In turn, the remodeled ECM can modify the gradients, bioavailability, and proteolytic activation of matrix-bound ligands [114,115]. Reviewing the vast number of signaling molecules that modulate VSMC’s function goes beyond the scope of this review; however, we will briefly summarize the main features of three pathways that have been specifically implicated in TAA pathogenesis: angiotensin II, TGF-β, and Notch signaling [116,117].

#### 3.2.1. Angiotensin II Signaling via Angiotensin II Type 1 Receptor

The renin–angiotensin system is a critical regulator of both physiological and pathological cardiovascular functions (comprehensively reviewed in reference [118]). Its primary effector ligand, angiotensin II, has both hormone-like and cytokine-like effects on VSMCs [119,120]. Angiotensin II binding to angiotensin II type 1 receptors (AT_1_Rs) activates signaling pathways that stimulate VSMC contraction within seconds, inducing vasoconstriction and regulating blood pressure [121]. Longer exposures to angiotensin II regulate VSMCs growth, hypertrophy, migration, and ECM deposition [122,123]. In addition, angiotensin II signaling via AT_1_R can promote vascular inflammation by the upregulation of adhesion molecules and leukocyte chemoattractants [124]. Some of the AT_1_R effects are mediated by the transactivation of receptors for other growth factors including those for PDGF and epidermal growth factor (EGF) [125,126,127]. AT_1_R signaling also increases the expression of other signaling molecules, including PDGF, transforming growth factor-β (TGF-β), and reactive oxygen species (ROS) [124,128,129,130].

In VSMCs, AT_1_R signaling mediates the deposition of collagens in response to mechanical stimulation through a process that depends on increased expression of the TGF-β ligand [129,131,132,133]. AT_1_R also potentiates TGF-β signaling by inducing the expression of metalloproteinase-2 and -9 (MMP-2 and MMP-9), which are proteolytic enzymes that can catalyze the conversion of inactive ECM-bound latent TGF-β into its bioactive form [134,135,136]. AT_1_R signaling, which can be triggered through mechanical stretch, independently of angiotensin II [137,138,139,140], has been shown to be overactivated in mouse models of TAA by several mechanisms [117]. These include the upregulation of *Agtr1a* (the gene that in rodents codes for the major form of AT_1_R expressed in the thoracic aorta) via the ROS-dependent activation of nuclear factor kappa B (NF-kB); the de-repression of *Agtr1a* expression as a consequence of defective TGF-β signaling; and increased expression of angiotensin-converting enzyme, which is a positive regulator of the pathway [141,142,143].

#### 3.2.2. TGF-β Signaling

TGF-β ligands (TGF-β1, TGF-β2, and TGF-β3) are secreted as inactive latent complexes that are activated by chemical (acidification), mechanical (integrins), and enzymatic (proteases) processes within the ECM [144]. After conversion to its active form, TGF-β ligand binds to a tetrameric receptor complex formed by TGF-β receptor I (TβRI) and TGF-β receptor II (TβRII), and it induces receptor-mediated phosphorylation of intracellular signaling mediators, which are mothers against decapentaplegic homolog 2 (Smad2) and 3 (Smad3) [145]. Phosphorylated Smad2 and Smad3 (p-Smad2/3) bind to the common-mediator Smad4, translocate to the nucleus, and regulate the transcription of TGF-β target genes [146]. The activation of Smad signaling induces the expression of inhibitory Smad proteins, Smad6 and Smad7, which suppress signaling in a negative feedback loop [147]. Additional repressors of TGF-β signaling include transcription co-factors Ski and SnoN, which negatively regulate signaling by disrupting the formation of complexes between Smad2/3 and Smad4, and by inhibiting their association with p300 coactivators [148,149,150,151].

Focal adhesion-dependent tension modulates TGF-β signaling both negatively and positively [152]. Focal adhesions can negatively regulate TGF-β signaling by physically separating TβRI, which is found within focal adhesions, from TβRII, which is more commonly located around the edges, thus preventing formation of the functional TβRI/TβRII signaling complex [153]. The inhibition of myosin-dependent force generation disrupts tension and results in re-assembly of the TβRI/TβRII receptor complex, allowing signal transduction [153]. Force generation through the actin–myosin contractile unit can also positively regulate TGF-β signaling by generating sufficient tension to promote the integrin-mediated release of bioactive TGF-β from its latent biologically inactive complex [154,155].

TGF-β signaling regulates many aspects of VSMC biology, including positive regulation of VSMC-specific transcripts such as *Myh11*, *Acta2*, and *Cnn1* (coding for SM-MHC, αSMA, and Calponin-1, respectively) [156,157,158]. Positive effectors of TGF-β signaling and TGF-β-responsive genes are upregulated in aneurysmal lesions of various etiology in both patients and mouse models, where they contribute to both adaptive and maladaptive responses [65,117,159,160,161,162]. TGF-β signaling also closely interacts with other pathways that regulate VSMC phenotypes, including AT_1_R and Notch signaling [54,163].

#### 3.2.3. Notch Signaling

Notch signaling is activated by engagement of a Notch receptor (Notch1 to Notch4) by one of its ligands (Jagged1/2 and Delta-like1/3/4 ligands) [116]. The binding of Notch to its cognate receptor induces a conformational change that triggers multiple proteolytic processing steps that release the Notch intracellular domain (NICD) from the membrane and promote its translocation to the nucleus, where it regulates transcription via interaction with other factors [164].

Adult VSMCs express Notch1, Notch2, and Notch3 receptors and the ligand Jagged-1. Notch2 and Notch3 appear to be the main receptors modulating phenotype and functions in VSMCs, but the activation of both Notch1 and Notch3 facilitates VSMC migration in vitro [165,166,167,168]. Signaling initiated by interactions between receptors and ligands expressed on neighboring VSMCs positively regulates the transcription of multiple transcripts including *Acta2* and Pdgfbr (coding for PDGF-β receptor) [168,169,170]. Whereas Notch positively regulates VSMC specification during development, its role in the modulation of adult VSMC phenotypes is less clear, having been shown to promote both “contractile” and “synthetic” phenotypes, depending on the study [168,171,172,173,174,175,176].

Notch signaling interacts with both angiotensin-II and TGF-β signaling. In podocytes, stimulation with angiotensin II upregulates *Notch1*, and inhibition of Notch1 decreases angiotensin-II dependent upregulation of *Tgfb1* while promoting upregulation of MMP-2 and MMP-9 [177]. Notch and TGF-β signaling cooperate to induce the expression of contractile proteins, including through the direct binding of Smad2/Smad3 to CBF1, which is a Notch-regulated transcription factor [174,178,179]. However, Notch signaling antagonizes TGF-β signaling by reducing the expression of TGF-β receptor II via the induction of miR145 [180]; in turn, TGF-β antagonizes Notch signaling by decreasing the expression of *Notch3* [181]. The expression of Notch ligands and receptors is dysregulated in TAA, and defective Notch signaling has been associated with TAA that develops in patients with malformations of the aortic valve [175,182,183].

## 4. Genes Associated with Syndromic and Non-Syndromic Hereditary Thoracic Aortic Aneurysm

The identification of genetic variants that cause hereditary forms of aneurysm provides the opportunity to clearly define the molecular deficiencies that initiate this disease. Several recent reviews have summarized the current list of approximately 30 genes involved in the development of either syndromic or non-syndromic forms of TAA [14,184,185,186] (Figure 1B). Although criteria for inclusion vary, 11 genes are currently confirmed as “definitive” determinants of highly penetrant TAA based on the Clinical Genome Resource framework [187]. Despite this progress, a causative mutation is found in only approximately 30% of patients with a clear positive family history, suggesting that causative mutations in genes not currently tested in familial cases of TAA remain to be identified [184].

Mutations that cause TAA interfere with the function of genes that encode components of the ECM or proteins implicated in the transduction of either mechanical or biochemical signals in VSMCs [14,184,185,186]. The list of known TAA-associated genes, mode of inheritance, the primary biochemical function perturbed by causal variants, and associated disease are described in Table 1. Detailed phenotypic features of syndromic and non-syndromic TAA disorders, and the implications for clinical management, have been described in detail elsewhere and will not be discussed here [6,13,184,185,186]. Instead, we discuss the basic molecular functions that are impaired by TAA-associated mutations and the mechanisms by which these deficiencies may initiate TAA pathogenesis.

### 4.1. Genes Coding for Components of the Extracellular Matrix

#### 4.1.1. Fibrillin-1

Several TAA-causing mutations are found in genes coding for structural components of elastic fibers or for proteins necessary for their assembly or maturation. *FBN1* codes for fibrillin-1, the primary component of microfibrils, which surround and connect elastic fibers to cellular integrins [80,233]. Heterozygous nonsense, missense, and complete deletions of *FBN1*, as well as mutations affecting splicing, cause hereditary aneurysms associated with MFS [188,234,235,236]. Loss-of-function mutations in *FBN2,* the gene coding for fibrillin-2, cause congenital contractural arachnodactyly; although these mutations are associated with aortic dilatation and dissection in some patients, TAA is not a common phenotype, which is likely a consequence of the compensatory effect of fibrillin-1 [200,201]

TAA-associated *FBN1* mutations result in defective synthesis, secretion, and/or the incorporation of fibrillin-1 in microfibrils [234,236,237,238]. In MFS mouse models, loss of fibrillin-1 results in defective connections between VSMCs and elastic lamellae, probably because of reduced integrin binding to elastin-associated microfibrils [80,239]. Mutations in *FBN1* have also been proposed to reduce the ability of fibrillin-1 to bind and sequester the latent TGF-β complex into the ECM, thus promoting its conversion into bioactive TGF-β and causing excess activation of this pathway [159,240,241]. However, whereas mice carrying MFS-causing mutations in fibrillin-1 develop TAA, mice expressing a mutant form of fibrillin-1 engineered to lack its TGF-β-binding domain do not [242,243]. This evidence suggests that the mechanism by which mutant fibrillin-1 leads to over-activation of TGF-β may be more indirect. Under the revised hypothesis, loss of connection between integrins and mutant fibrillin-1 initiates phenotypic changes in VSMCs that culminate in the induction of MMPs and, consequently, the proteolytic activation of latent TGF-β [239,243,244,245].

#### 4.1.2. Lysyl Oxidase

Heterozygous inactivating mutations in the *LOX* gene, which encodes lysyl oxidase, cause aneurysms in both patients and animal models [197,198,246]. Lysyl oxidase catalyzes the oxidation of lysine residues and crosslinking reactions necessary for the assembly and stabilization of both collagen and elastin [247,248,249]. In consequence, patients and mouse models carrying mutations that reduce the levels or activity of this enzyme show impaired assembly of collagen and elastic fibers in the aortic wall [197,198,246]. Lysyl oxidase has also been proposed to regulate cell signaling by the direct modification of cell surface receptors and ligands [250,251]. Although direct oxidative deamination of lysine residues in TGF-β1 was not proven, lysyl oxidase binds to TGF-β1 in vitro, and genetic or pharmacological inhibition of lysyl oxidase activity in cell culture results in increased responsiveness to TGF-β, as measured by the induction of pSmad3 and Smad-dependent transcriptional activity [252,253]. It is not known if the impairment of these latter functions contributes to the mechanism by which *LOX* mutations cause TAA [254,255].

#### 4.1.3. Fibulin-4

Phenotypes similar to those caused by deficiency in lysyl oxidase activity are observed as a consequence of homozygous loss-of-function variants in *EFEMP2,* the gene coding for fibulin-4, a matrix glycoprotein. These mutations are predicted to interfere with fibulin-4-mediated enhancement of lysyl oxidase enzymatic activity and/or recruitment to immature elastin molecules [248,256,257]. Accordingly, fibulin-4 deficiency results in disorganized collagen and elastin fibers in the aortic wall as well as aneurysms of the ascending aorta in both patients and mouse models [248,258,259]. Fibulin-4-deficient mouse models develop ascending aortic aneurysms in association with loss of connections between the elastic fibers and VSMCs, and secondary upregulation of angiotensin II signaling [260,261]; this process was shown to depend on increased sensitivity of fibulin-4-deficient VSMCs to mechanical stretch, which causes an upregulation of thrombospondin-1—a matrix glycoprotein that negatively regulates cell adhesion and positively regulates both angiotensin II and TGF-β signaling [261]. Germline genetic inactivation of thrombospondin-1 in fibulin-4-deficient mice improved the appearance of elastic fibers in the aorta and restored connections between the elastic lamina and VSMCs, suggesting that in these mouse models, pathogenesis was initiated by disruption of the elastin-contractile units and aggravated by downstream upregulation of factors, such as thrombospondin-1, that further compromise the establishment of ECM–VSMC contacts [261].

#### 4.1.4. Microfibril-Associated Glycoprotein 2

Loss-of-function mutations in *MFAP5*, which codes for microfibril-associated glycoprotein 2 (MAGP2) [196], also predispose to development of TAA. MAGP-2 (and MAGP-1) are matrix glycoproteins that interact with fibrillin-1 and modulate both TGF-β and Notch signaling [262,263,264]. However, the mechanism by which mutations in these genes initiate disease is not fully understood [263].

#### 4.1.5. Biglycan

X-linked inactivating mutations in the *BGN* gene, which codes for the ECM protein biglycan, cause syndromic forms of TAA [195]. Biglycan is a small leucine-rich proteoglycan (SLRP) that interacts with, and regulates the function of, several matrix proteins, including collagen and TGF-β [265,266,267,268]. Biglycan-deficient male mice die from aortic rupture; analysis of aortic tissue shows the presence of smaller collagen fibrils, leading to the hypothesis that a defective collagen structure reduces the resistance of the aorta to passive stress [269,270]. Supplementary to its structural roles, biglycan also binds and sequester TGF-β in the ECM, acting as a negative regulator of TGF-β activation [271,272]. Its deficiency may thus increase TGF-β bioavailability through a mechanism similar to what was originally proposed for MFS-causing mutations in *FBN1* [195,270].

#### 4.1.6. Collagen Type III α 1 Chain

Mutations in the collagen gene *COL3A1* are associated with Ehlers–Danlos syndrome (EDS) [193,194], which is a syndrome associated with spontaneous arterial dissection and/or rupture in the absence of preceding aneurysmal dilatation [192,193,194]. These mutations interfere with the maturation and deposition of collagen, suggesting that this molecule serves a protective function against catastrophic failure of the aortic wall [255,269,273]. Notably, work performed in mouse models of EDS suggests that the protective role of collagen might go beyond its structural function and encompass the indirect regulation of signaling mechanisms that can be targeted pharmacologically to reduce the rate of rupture and death [274].

#### 4.1.7. Tropoelastin

Rare mutations in *ELN*, the gene coding for tropoelastin, cause autosomal dominant Cutis Laxa (ADCL), which is a condition that is associated with aortic dilatation and rupture in approximately 30–50% of patients [85,199,275]. Most of the genetic abnormalities in ADCL are frameshift mutations that result in an elongated tropoelastin protein, which forms larger aggregates with the reduced ability to bind microfibrils [276]. These mutations are proposed to cause abnormal deposition and the assembly of elastic fibers via a dominant negative effect; they may also cause endoplasmic reticulum stress and apoptosis through the accumulation of misfolded tropoelastin [85,277,278].

Whereas dysfunctional elastin has been associated with TAA, elastin insufficiency associates with inherited obstructive arterial diseases; heterozygous loss-of-function mutations in *ELN*, including deletion of the whole gene, cause supravalvular aortic stenosis and Williams Beuren Syndrome, but not aneurysm [85,279,280,281,282,283,284,285]. Similarly, *Eln^-/-^* mice, which completely lack elastin, die of obstructive arterial disease, not dissection, a few days after birth [283]. This is in contrast with *Lox^-/-^* mice, which lack lysyl oxidase, and which also die perinatally, but show large aortic aneurysms [246]. More recently, VSMC-specific elastin deficiency was shown to result in almost a complete depletion of elastic lamina in the arterial wall, lengthening and thickening of the ascending aorta, mild luminal obstruction, aortic coarctation, and secondary cardiomyopathy, but not dilation or aneurysm [286]. Mouse models with only one functional *Eln* allele are hypertensive and have a paradoxically increased number of elastic lamellae, but they show no overt signs of degenerative vascular disease, and again, no aneurysm or dissection [287,288]. Although the limited lifespan of elastin-deficient mice prevents their long-term observation, complete loss of elastic lamellae—and thus of the elastin-contractile unit—does not appear to be sufficient to initiate the development of aneurysm. Similarly, patients with homozygous loss-of-function mutations in *FBLN5,* the gene coding for fibulin-5, develop autosomal recessive Cutis Laxa type IA (ARCL1A). Deficiency in fibulin-5, a matrix protein that assists in the deposition of tropoelastin on microfibrils, results in impaired elastic fiber assembly and fragmentation of elastic laminae in the aorta, but there is no evidence of aneurysms or dissections in either patients or mouse models [276,289,290,291,292]

Taken together, this mutational repertoire strongly suggests that interference with the assembly and function of elastic lamellae, and therefore impairment of the elastin-contractile unit, is an initiating event in aneurysm pathogenesis [82,293]. However, the fact that mere loss of elastin is insufficient to cause TAA suggests that additional mechanisms actively causing dysfunctional signaling downstream of integrins and focal adhesion may be necessary to trigger aortic dilation.

### 4.2. Genes Coding for Proteins Involved in Transduction of Mechanical Signals

#### 4.2.1. Smooth Muscle Specific Contractile Proteins, α-SMA and SM-MHC

Mutations in genes involved in the structure or regulation of actomyosin filaments cause familial cases of TAA. These include mutations in *ACTA2,* the gene coding for α-SMA, and in MYH11, which codes for SM-MHC [217,218,294,295,296]. Mutations in these genes are predicted to result in decreased ability of the corresponding monomeric protein to assemble into polymeric filaments, thus impairing the function of the actin–myosin contractile unit [62,217,218,294,295,296].

#### 4.2.2. Myosin Light Chain Kinase and cGMP-Dependent Protein Kinase

TAA-causing mutations are also found in genes involved in the regulation of myosin-dependent contraction [87]. TAA-associated mutations in *MYLK,* which codes for MLCK, reduce its kinase activity and thus impair contraction and promote relaxation [219,297,298,299]. Similarly, one recurring TAA-associated gain-of-function mutation (pR177Q) in *PRKG1*, which codes for type I cGMP-dependent protein kinase (PKG-1), results in the constitutive activation of PKG-1, a cGMP-activated enzyme that promotes relaxation by phosphorylating and activating MLCP [87,220].

#### 4.2.3. Filamin-A

X-linked loss-of-function mutations in the *FLNA*, which codes for filamin A, result in an increased risk of TAA, among other symptoms [221,222,224,300]. Filamin A is a cytoskeletal regulatory protein with multiple functions, including as an anchoring protein within focal adhesions, as a linker between actin filaments, and as a scaffold for many signaling pathways [301,302,303,304]. It is not clear which of the many functions of filamin A is critical to the prevention of TAA [224].

#### 4.2.4. Ari-1

Rare variants in *ARIH1*, which codes for Ariadne drosophila homolog 1 (Ari-1), are found in individuals with aortic or cerebrovascular aneurysms [225]. Ari-1 is a component of the LINC complex, and VSMCs derived from patients carrying these mutations show aberrant nuclear morphology in culture [225]. The existence of these mutations suggests that defective LINC complex function, which links actomyosin function to nuclear and epigenetic regulation, may play a role in aneurysm development [111,112]. Recently, histone deacetylase 9 (HDAC9) was found to be upregulated in aortic tissue samples from syndromic, familial, and sporadic cases of TAA, and to be involved in epigenetic silencing of cytoskeletal and contractile proteins [305]. The defective epigenetic regulation of gene expression by histone modifications, deoxyribonucleic acid (DNA) methylation, and noncoding ribonucleic acids (RNA) has been shown to be involved in TAA pathogenesis and is reviewed in ref. [306].

The fact that mutations in genes that control the function of actin–myosin complexes cause TAA clearly points to the importance of force generation to the homeostasis of the aortic wall [87]. Together with mutations that affect elastic fiber assembly, these mutations strongly suggest that dysfunctional connections between ECM, integrins, and cytoskeleton initiate the pathogenic process in TAA [82,87]. However, it remains unclear which of the numerous mechanical functions served by the actin–myosin contractile unit, which is involved in the force-dependent regulation of biochemical signals and transcriptional programs, is most crucial to preventing pathogenic phenotypic changes in VSMCs [53].

### 4.3. Genes Coding for Proteins Involved in Transduction of Biochemical Signals

#### 4.3.1. Positive Regulators of the TGF-β Signaling Pathway

Heterozygous loss of function mutations in genes coding for positive regulators of TGF-β signaling, including receptors (*TGFBR1*, *TGFBR2*), transduction molecules (*SMAD3* and *SMAD2*), and ligands (*TGFB2* and *TGFB3*) cause TAA associated with LDS [202,203,204,205,206,207,307,308,309,310,311]. Additionally, individuals with compound heterozygous or homozygous variants in *LTBP3*, the gene encoding latent TGF-β binding protein 3 (LTBP3), suffer from aneurysms and dissections of the thoracic aorta and other arteries [210]. Although LTBP3 maintains TGF-β into its latent state, and thus could be considered a negative modulator, it is also necessary for its secretion, and thus, LTBP3 deficiency is associated with impaired TGF-β signaling [312]. This mutational repertoire clearly indicates that a decrease in canonical TGF-β signaling initiates the pathogenic process that culminates in the development of aneurysm. However, the exact pathogenic mechanisms, and the process by which it leads to aneurysmal lesions invariably associated with increased or, in some cases, unchanged, TGF-β activity (as measured by levels of pSmad2/3 and expression of target genes), remains unclear [142,202,203,204,205,206,207,308,309,310,311,313,314,315].

Regardless of the specific mechanism, most models have envisioned an initial phase of defective TGF-β signaling followed by a phase of increased signaling, which is associated with excess activation of AT_1_R signaling. However, models diverge regarding the mechanisms that trigger the transition from a “low” to a “high” TGF-β state. One model suggests that reduced TGF-β signaling impairs the expression of critical contractile proteins, thus causing defects in the elastin-contractile unit that trigger the upregulation of angiotensin II and TGF-β signaling as part of an “injury” response [5,41]. As discussed, TGF-β positively regulates the expression of contractile proteins, and therefore, the effect of defective TGF-β signaling could resemble that of mutations impairing α-SMA or SM-MHC function [141,156,307,316]. However, decreased expression of contractile proteins is not uniformly detected in aneurysmal tissue or in VSMCs from LDS patients and mouse models, or upon postnatal genetic inactivation TGF-β signaling, with studies reporting both increased or decreased expression [313,315,317,318,319].

Another model proposes that LDS mutations, which impair but do not fully abolish signaling, have an uneven effect on the signaling capacity of VSMCs of different embryonic lineages (and potentially on that of different cell-types), and drive excess signaling thorough mechanisms that depend on this imbalance [142,320]. The proximal thoracic aorta is populated by VSMCs derived from cardiac neural crest (CNC) [321] and second heart field (SHF)-derived progenitors [322,323]. In mouse models of LDS carrying a kinase-inactivating mutation in *Tgfbr1* (*Tgfbr1^M318R/+^*), SHF-derived VSMCs showed the expected deficit in responsiveness to TGF-β, whereas CNC-derived VSMCs retained normal signaling capacity [142]. This associated with the upregulation AT_1_R-dependent TGF-β ligand expression in SHF-derived VSMCs and an excessive activation of TGF-β signaling in surrounding CNC-derived VSMCs [142,320]. Interestingly, studies of human TAA samples have observed that an increased storage of TGF-β1 is not uniform across the media thickness and tends to accumulate in the outer third of the media, closer to the adventitial layer, where SHF-derived cells are predicted to reside [65,323,324].

Work conducted in induced pluripotent stem cells (iPS)-derived VSMCs has confirmed that lineage-of-origin modifies the effect of LDS-causing mutations, impairing signaling in SHF-like VSMCs derived from cardiovascular progenitors, while leaving the signaling capacity of CNC-derived VSMCs intact or increased [325]. Only SHF-like VSMCs, but not CNC-derived VSMCs, had reduced the expression of genes that promote a contractile phenotype [325]. Taken together, these data suggest that the two models might be reconciled by hypothesizing that SHF-derived VSMCs are the cells that primarily undergo phenotypic switching, downregulating the expression of contractile proteins, and upregulating AT_1_R-dependent secretion of TGF-β, thus causing increased signaling in neighboring CNC-VSMCs [325].

#### 4.3.2. Negative Regulators of the TGF-β Signaling Pathway

Mutations in genes coding for negative regulators of the TGF-β pathway have been associated with syndromic forms of aneurysm. As discussed earlier, aneurysms can be caused by inactivating mutations in the *BGN* gene, which can function as a negative regulator of TGF-β signaling [195,271,272]. Additionally, Shprintzen–Goldberg syndrome (SGS) is caused by mutations in the *SKI* gene proposed to interfere with SKI’s ability to bind and inactivate the transcriptional activity of Smad proteins [149,211,212,326]. More recently, however, these mutations have been shown to stabilize the SKI protein, causing attenuation rather than enhancement of TGF-β signaling as originally proposed [327]. 

#### 4.3.3. Notch1

The perturbation of Notch signaling, a pathway that can both cooperate and antagonize TGF-β signaling during VSMC differentiation, increases susceptibility to aortic aneurysm [174,175,176]. Although the mechanisms remain unclear, loss-of-function mutations in *NOTCH1* predispose to hereditary forms of aneurysms that associate with aortic valve defects [213,214,215,216,328]. The effect of these mutations is often variable and may be modified by the presence of additional predisposing factors [175]. In mouse models, *Notch1* haploinsufficiency causes aortic dilation in mice of a permissive genetic background (129S6) and exacerbates aortic root pathology of MFS mouse models, independently of aortic valve defects [329]. Although the mechanisms by which loss of Notch signaling causes TAA remain unclear, defective developmental interactions between endothelial cells and VSMCs, and direct effects on VSMC phenotype have both been proposed [175,183,330]. Notably, whereas Notch signaling appears to be protective in TAA, it tends to exacerbate inflammation-driven AAA [175,331,332].

### 4.4. Genetic Variants Associated with Bicuspid Aortic Valve (BAV) with Aneurysm

Bicuspid aortic valve (BAV) is a common congenital defect that can be present in isolation or in association with other syndromic manifestations, and which associates with an increased risk of aortic aneurysm, frequently affecting the ascending aorta [2,6,117,333]. Although the genetic architecture of BAV is complex, increased risk for BAV is generally inherited in an autosomal-dominant manner, with variable expressivity and incomplete penetrance [334]. Genetic abnormalities associated with an increased risk for BAV with aneurysm include those that cause syndromes such as LDS and Turner syndrome [334], and TAA-associated mutations in genes such as *NOTCH1*, *ELN*, *ACTA2*, *FBN1,* and *LOX* (discussed above), all of which, however, explain a very small portion of all BAV cases [213,217,236,277,335,336,337]. Genetic variants associated with BAV have also been identified in genes coding for transcription factors of the GATA family [338,339,340,341], modulators of the Bone Morphogenic Protein (BMP) signaling pathway [342,343,344], members of the A Disintegrin And Metalloproteinase With Thrombospondin Motifs (ADAMTS) family of multidomain extracellular proteases [345,346], and the transcription factor TBX20 [347]. Recently, mutations in the gene *ROBO4*, which codes for Roundabout homolog 4 (ROBO4), have been identified in patients with BAV and aneurysm of the ascending aorta [348]; these mutations are predicted to interfere with ROBO4′s function in the modulation of endothelial to mesenchymal transition [348]. Although aneurysms of the ascending aorta associated with BAV share features such as medial degeneration and VSMC phenotypic abnormalities with other forms of TAA, their pathogenesis is partly distinct, might involve a greater contribution of endothelial cell dysfunction and blood flow disturbances [47], and is reviewed in detail in references [349,350,351,352].

## 5. Proposed Model of TAA Pathogenesis Based on the Function of Known Causal Variants

**Figure 2:**
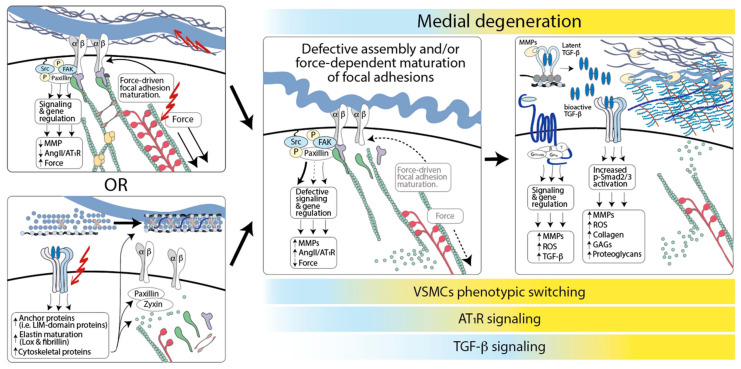
A model for hereditary aneurysm pathogenesis. Defective assembly of connections between elastic fibers and VSMCs initiates their phenotypic switch toward a proteolytic phenotype that promotes medial degeneration. The pathogenic process can be initiated by genetic mutations (red lightening) that directly target ECM structures or intracellular components necessary for the assembly/function of focal adhesions, including cytoskeletal components necessary for force-dependent focal adhesion maturation. It can also be initiated by mutations that target signaling pathways, such as TGF-β and Notch signaling, which modulate the expression of components of the focal adhesion machinery (ECM components, adaptor/anchoring proteins such as zyxin and paxillin, and actin-binding proteins such as Sm22). Opportunity to establish proper connections between VSMCs and the ECM may be developmentally restricted, given that adult VSMCs have limited ability to elaborate and organize components of the elastic fibers. Dysfunctional signaling downstream of focal adhesions would promote phenotypic switching, inducing a downregulation of contractile proteins, upregulation of metalloproteinases, and other matrix proteins, such as thrombospondin-1, that positively regulate AT_1_R and TGF-β signaling, further exacerbating ECM pathogenic remodeling. The activation of AT_1_R signaling and AT_1_R-dependent upregulation of TGF-β signaling would result in the deposition of collagen, accumulation of proteoglycans, and production metalloproteinases and reactive oxygen species (ROS). TGF-β signaling is modeled as protective at early stages of disease, when it may promote the restoration of ECM–VSMC interaction, and progressively more pathogenic when the establishment of ECM–VSMC interaction is no longer developmentally possible, and TGF-β promotes the deposition of proteoglycans and glycosaminoglycans, and production of ROS.

The discovery of mutations that cause hereditary forms of TAA has identified fundamental cellular and molecular process that are necessary to retain VSMC homeostasis and prevent medial degeneration. The preponderance of evidence supports a model whereby a defective assembly of connections between the ECM and VSMCs initiates the phenotypic transition of these cells from a quiescent, contractile phenotype to one that is highly synthetic and conducive to proteolytic degradation of the ECM [5,53,353] (Figure 2). Experiments in mouse models of TAA associated with fibrillin-1 and fibulin-4 deficiency indicate that defective connection of VSMCs to the elastic laminae is an early event in pathogenesis, which is followed by the downregulation of contractile proteins, upregulation of metalloproteases, and induction of signaling pathways that further promote phenotypic switching and matrix degradation [239,261]. Accordingly, single-cell RNA sequencing (scRNA-Seq) analysis of aortic tissue from a mouse model of MFS shows that although the expression of transcripts coding for contractile proteins such α-SMA, SM-MHC, and Calponin-1 is unchanged at early stages of the disease, the expression of these markers progressively decreases in association with the upregulation of AT_1_R and TGF-β signaling in a specific subset of VSMCs [239,244,354].

However, a loss of VSMC-elastic fibers connections does not appear to be sufficient to cause aneurysmal disease, given that elastin deficiency associates with obstructive arterial diseases and VSMC over-proliferation, but not aneurysm [85,293]. TAA-causing mutation may result in the abnormal assembly, maturation, and function of focal adhesions and actively perturb focal adhesion- and force-dependent signaling in a manner that is not recapitulated by simple loss of attachments to elastin fibers [28,87,91,355]. Then, dysfunctional signaling downstream of focal adhesions would promote the upregulation of metalloproteinases and other matrix proteins, such as thrombospondin-1, which positively regulate AT_1_R and TGF-β signaling, further exacerbating ECM pathogenic remodeling [239,244,261,354]. Although the direct potentiation of TGF-β receptor activity by interference with myosin-driven focal adhesion maturation has been shown in vitro, it is not known whether it occurs in vivo [152,153].

In this model, mutations in components of matrix proteins or enzymes necessary for their assembly/maturation would initiate disease by directly compromising the function of elastic fibers or other ECM structures to which integrins connect, whereas mutations in components and regulators of the actin–myosin cytoskeleton would do so by perturbing tension and/or myosin-dependent maturation of focal adhesions [62,141,217,218,219,239,261,294,295,296,297,298,299].

Mutations in components of signaling pathways that play a positive role in VSMC differentiation, such as TGF-β and Notch signaling, would initiate disease indirectly by interfering with the expression of components required for the assembly of functional focal adhesions. For example, TGF-β signaling controls the assembly of elastic fibers by regulating the expression of fibrillin-1, elastin, and lysyl oxidase; it also modulates the expression of proteins involved in focal adhesion and cytoskeletal rearrangement such as paxillin, Sm22 (Transgelin), and Lin11, Isl-1, and Mec-3 (LIM) domain-containing proteins CRP2 and zyxin [356,357,358,359,360,361,362,363,364,365]. Once lost, the opportunity to establish proper VSCM–ECM connections may be lost forever, given that adult VSMCs have a limited ability to elaborate and organize components of the elastic fibers [23,293]. As a result, once dilation or aneurysm is detectable, the activation of adaptive and maladaptive pathways in response to the original molecular defect would be ongoing.

Current therapeutic strategies focus on decreasing the rate of dilation, and the associated risk for acute aortic events, by reducing hemodynamic stress on the vessel wall with anti-hypertensive drugs, such as inhibitors of the β-adrenergic receptor [366]. A better understanding of adverse and beneficial signaling pathways activated in response to the primary genetic insult might allow the development of therapies that attempt to disentangle homeostatic and adaptive processes from maladaptive responses [367]. In this context, interventions aimed at reversing pathogenic transcriptional signatures that are epigenetically “locked in”, such as defective expression of Sm22, might prove beneficial [305,368].

## 6. Adaptive and Maladaptive Responses in TAA: Implications for Therapy

We have a limited understanding of the compensatory mechanisms activated in response to germline TAA-associated mutations. Feedback responses attempting to offset the negative consequences of a given genetic variant are active throughout prenatal and postnatal development and might significantly modify the structural, cellular, and molecular properties of the adult aorta. Mechanisms that are adaptive early, such as the activation of secondary pathways that compensate for the initial deficiency, might become maladaptive later on due to divergent effects on adult versus embryonic tissues, over-activation, or secondary activation of deleterious pathways. This might be especially true for mutations that impair signaling involved in morphogenesis, such as TGF-β and Notch signaling, given that the mitigation of defective signaling in these pathways is a condition necessary for the development of vascular structures and survival [175,369,370]. Additionally, while it is tempting to assume that all the phenotypic changes observed in TAA at the structural, cellular, and molecular level are contributing factors to disease, some may represent ongoing beneficial compensatory responses.

### 6.1. Adaptive and Maladaptive Roles of “Aortic Stiffness”

Loss of elastin and the increased deposition and crosslinking of collagen during aneurysm development translate into biomechanical changes that include reduced distensibility and increased stiffness of the aorta [71]. Changes in stiffness modulate VSMCs phenotypes through integrins and focal adhesions; although stiffness is generally associated with the retention of a “contractile” phenotype, excess stiffness can also increase sensitivity to growth factors, such as PDGF, which promotes a “synthetic” phenotype, and enhanced ECM stiffness has been shown to promote a switch from a “contractile” to a “synthetic” phenotype through the downregulation of DNA methyltransferase 1 [355,371,372,373].

Perhaps unintuitively, increased stiffness (resistance to deformation) can associate with decreased vessel strength (ability to withstand stress without breaking), with one study measuring an approximately 30% decrease in vessel strength accompanied by a 72% increase in stiffness in aneurysmal versus nonaneurysmal ascending aorta [374,375]. Correlations between increased stiffness and aortic dilatation have been reported in numerous studies of both patients and mouse models of TAA [376,377,378,379,380,381,382,383,384,385,386,387,388,389,390].

Although measures of distensibility and circumferential strain lose predictive power once aortic diameter is included in the analysis, a recent study of one hundred and seventeen MFS patients showed that measurements of longitudinal strain in the proximal aorta was a predictor of adverse aortic events (such as elective aortic root surgery or dissection), thus providing support to the notion that aortic stiffness could be considered for the stratification of patients based on risk [376,391,392].

These observations may suggest that increased collagen deposition and crosslinking is uniformly deleterious in TAA. However, other studies have shown that collagen deposition, especially in the adventitial layer, can be protective and part of beneficial “scar repair” mechanisms preventing transmural ruptures [71,375,393,394]. Consistent with these latter observations, genetic or pharmacological inactivation of lysyl oxidases, enzymes necessary for collagen and elastin cross-linking, cause or exacerbate aneurysm in patients and animal models [197,198,246,255,269,273,395,396,397]. The detrimental effects of fluoroquinolones on TAA pathogenesis have also been attributed to excess ECM degradation and reduced levels of collagen [398,399,400,401].

Taken together, these data suggest that the deposition of properly crosslinked collagen confers strength to the vessel, thus protecting it from mechanical failure. However, its effect might be highly dependent on the type and quality of collagen and the effect of ECM stiffness on VSMC phenotypes [71,402,403,404,405]. In vitro experiments in which levels of stiffness can be experimentally modulated show that both overly soft and overly stiff substrates fail to support functional focal adhesions and actin–myosin dynamics, and that nanoscale level patterning of substrata that mimics physiological conditions can modulate the effect of stiffness; VSMCs grown on nanopatterned soft substrata had a higher expression of VSMC markers associated with a quiescent, contractile phenotype (smoothelin, calponin-1), and lower expression of inflammatory markers (monocyte chemoattractant protein-1) relative to nanopatterned “stiff” substrata, suggesting that matrix architecture and mechanics have combinatorial effects on VSMC mechanosensing and differentiation pathways [402,403,405].

### 6.2. Adaptive and Maladaptive Roles of TGF-β Signaling

Work performed in animal models clearly shows that TGF-β signaling is essential for aortic development and morphogenesis [406,407]. Additionally, the ablation of TGF-β signaling in VSMCs by the genetic inactivation of *Tgfbr2* postnatally results in aortopathy and dissections as well as an exacerbation of pathology in mice with a pre-existing genetic predisposition to aortic aneurysm, suggesting that postnatal aortic VSMCs require a basal level of TGF-β signaling for homeostasis [317,318,408]. Moreover, as discussed, heterozygous, inactivating mutations in positive effectors of this pathway cause hereditary forms of TAA [202,203,204,205,206,207,307,308,309,310,311]. In consequence of these observations, the increased levels of TGF-β ligand and nuclear pSmad2/3 observed in aneurysmal tissue obtained from patients and models carrying these mutations has been proposed to be part of a “repair” response [5,41].

Beneficial roles of TGF-β in TAA may include the suppression of AT_1_R signaling, induction of protective factors such as nexin-1 and proteases inhibitors, and promotion of contractile proteins expression [317,409,410,411,412,413]. In addition, TGF-β-dependent induction of collagen, lysyl oxidases, and other pro-fibrotic factors might contribute to thickening of the adventitial layer, which, as discussed, can be protective [71,269,364,414,415,416,417]. On the other hand, maladaptive effects of excess TGF-β signaling include an induction of glycosaminoglycans and proteoglycan accumulation within the arterial wall, upregulation of proteolytic enzymes that exacerbate ECM destruction, and stimulation of ROS production thorough several mechanisms, including by upregulation of NADPH oxidases (Nox) [46,418,419,420,421,422,423,424,425,426,427,428].

Accordingly, in contrast to the complete inactivation of TGF-β signaling in VSMCs, which unvaryingly enhances pathogenesis, the effect of partial TGF-β antagonism with neutralizing antibodies or by the inactivation of Smad proteins in selected cellular subsets is varied. Systemic TGF-β neutralization had no effect on angiotensin II-induced TAA [429], and it either had a beneficial or dimorphic effect in mouse models of MFS, with perinatal and postnatal antagonism being detrimental and beneficial, respectively [159,430,431]. In a recent study in a MFS mouse model, the beneficial effect of TGF-β neutralizing antibodies was correlated with a reduced expression of Nox4, restoration of normal levels of dihydrofolate reductase, and reduced levels of ROS production [431]. Additional studies in mouse models of hereditary TAA have shown that while germline *Smad4* haploinsufficiency is deleterious in MFS, *Smad2* deletion in CNC-derived VSMCs is beneficial in a mouse model of LDS [142,432]. Taken together, these data indicate that TGF-β signaling can serve both protective and pathogenic roles in TAA depending both on cell type and stage of disease [35,433].

### 6.3. Adaptive and Maladaptive Roles of Angiotensin II Signaling

In contrast with direct TGF-β antagonism, treatment with antagonists of AT_1_R signaling, such as the angiotensin receptor blocker (ARB) losartan, invariably prevents the development of aneurysm in animal models; this beneficial effect associates with reduced levels of TGF-β ligand, pSmad2/3, and the expression of TGF-β target genes [117,143,159,313,434,435,436]. In addition, the deletion of *Agtr1a* (gene encoding AT_1_R in mice) prevents aortic root dilation in two different MFS mouse models [437,438]. The beneficial effects of AT_1_R antagonism in mouse models of TAA have been attributed to its anti-hypertensive effects and to the inhibition of fibrotic, hypertrophic, and mitogenic responses activated by TGF-β and mitogen-activated protein kinase (MAPK) signaling [159,258,432,439]. Additionally, losartan treatment could reduce the AT_1_R-dependent secretion of glycosaminoglycans, whose accumulation has deleterious effects on the aortic wall [46,440]. Based on this evidence, several clinical trials have been initiated to test the efficacy of AT_1_R antagonism in the treatment of aneurysm in MFS patients. Although the degree of efficacy varied, none of the studies could replicate the remarkable beneficial effects routinely achieved in pre-clinical models; in one trial, the rate of adverse events was higher in the losartan-treatment group than in those receiving a hemodynamically equivalent dose of anti-hypertensive medication [441,442,443,444,445,446,447,448,449,450].

These results raised the possibility that AT_1_R signaling might have protective effects in TAA, both directly and through the enhancement of any protective effects of TGF-β signaling [367,451]. For example, the inhibition of AT_1_R-dependent collagen deposition and maturation, directly or through TGF-β-dependent pathways, could recapitulate, in part the detrimental effect of lysyl oxidase inhibition with β-amino-propionitrile (BAPN), which causes dissection or rupture in a number of animal models [255,395,396,397,414,415,417,452,453]. Additionally, AT_1_R-dependent signaling could be beneficial through stimulation of VSMC contraction [367,454]. Although the potential for beneficial effects of AT_1_R signaling need to be considered, other reasons may account for different outcomes in clinical trials relative to mouse models [117,455].

In mouse trials, losartan has been tested primarily in prevention rather than treatment of aneurysm, given that in most studies, the drug is initiated early, before overt disease is apparent; this is not the case for clinical trials, given that many patients have established aneurysms when treatment is started [456]. Additionally, in mouse trials, this drug is administered continuously throughout the day in drinking water or by an osmotic pump, at doses of approximately 50-100 mg/Kg/day; this is relevant because losartan has a short half-life of approximately 2 h [456] and the human equivalent dose [457] for the one used in mice would be 4–8 mg/Kg/day, which is much higher than the 0.4 to 1.5 mg/Kg/day used in clinical trials. In view of these considerations, it is notable that a recent double-blind, placebo-controlled, randomized clinical trial testing the effect of higher doses of irbesartan, an ARB with a longer half-life than losartan, significantly reduced aortic root dilatation in MFS patients [458].

The relative low frequency of adverse aortic events (dissection, rupture, death) makes it difficult to perform clinical trials with sufficient power to assess the effect of treatment on these clinically relevant outcomes, and most trials rely on the measurement of aortic size or growth to assess efficacy. Despite the statistical limitations, the most recent metanalysis of available data showed that AT_1_R antagonism slows the progression of aortic root dilation and is not associated with a statistically significant difference in adverse aortic events [366,459]. Additionally, a recently published long-term follow-up of the multicenter COMPARE trial, which originally reported a small but significant reduction in aortic root dilatation [450], showed that losartan treatment in MFS patients was associated with a decreased number of adverse aortic events in the treatment group [460]. Although more studies with sufficient power to confirm this study are both needed and planned, losartan is currently considered an acceptable treatment in combination with β-adrenergic receptor blockers, or when the latter are not tolerated [117,455].

## 7. Regional Heterogeneity within the Aorta

Although the composition of the aorta appears grossly uniform across the length of the vessel, different segments vary in structural, mechanical, cellular, and developmental properties. This regional heterogeneity reflects distinct physiological requirements for each aortic segment and might underlie the uneven distribution of aneurysm risk observed in patients carrying TAA-causing mutations, which preferentially tend to develop disease in the proximal aorta [461].

### 7.1. Developmental and Phenotypic Heterogeneity

Heterogeneity along the length of the aorta has been reported in regard to the expression of gap junction proteins, MMPs, homeobox transcription factors, and embryological origin [462,463,464,465,466,467,468]. The thoracic aorta develops with contributions from progenitors derived from the cardiac neural crest (CNC) and the mesoderm [321,469,470,471]. The pharyngeal portion of the splanchnic mesoderm gives rise to second heart field (SHF)-derived VSMCs, which populate the media of the aorta from the aortic root to the branching point of the innominate artery [322,323,472,473]. CNC-derived VSMCs are present from the aortic root to the end the aortic arch, where their contribution abruptly ends to that of VSMCs derived from the paraxial mesoderm [322,323,474,475]. In areas of overlap, CNC-derived VSMCs tend to line the lumen of the vessel, whereas SHF-derived VSMCs predominate on the adventitial side [323].

Lineage-dependent differences are retained in cultured VSMCs with respect to the expression of extracellular matrix proteins and contractile proteins, modulators of angiotensin II signaling, and responsiveness to TGF-β [476,477,478,479,480,481]. Although regional differences also apply to endothelial cells, the role of embryonic origin is less clear [482,483,484,485,486]. More recently, lineage-specific differences have been observed in iPS-derived VSMCs differentiated from mesoderm and neural crest progenitors [467,487,488,489,490]. iPS-derived VSMCs of mesoderm or CNC origin differ in phenotypes associated with MFS, LDS, and other forms of hereditary aortopathy [142,325,491,492]. As discussed, these differences might explain the unintuitive increase of TGF-β signaling observed in LDS patients and mouse models, and they may contribute to increased risk of dilation in the proximal aorta [142,325]; they might also explain discrepancies among laboratories in regard to the expression of contractile markers, with different studies reporting increased, and others reporting decreased, expression in TAA, even when using in situ methods that would not be confounded by the unequal representation of non-smooth muscle cell types among samples [307,368,493,494,495,496]. Additionally, the fact that dissections of the descending thoracic aorta often initiate in the region corresponding to the aortic isthmus might relate to the sharp transition between CNC-derived and paraxial mesoderm-derived VSMCs in this location, and the intrinsic difference in expression of metalloproteases and metalloprotease inhibitors in response to inflammatory mediators in these two lineages [467].

Although we lack markers to identify different VSMC lineages in human samples, the heterogeneity of VSMC phenotypes in TAA has been highlighted by scRNA-Seq analysis of human aortic samples from controls and patients with non-syndromic forms of TAA. This study identified at least four subtypes of VSMCs, including a proliferating VSMC cluster that expresses both synthetic and contractile marker genes, which is a phenotype that does not align with the traditional binary understanding of either “contractile” or “synthetic” phenotypes [497]. Although differences in the expression of contractile genes in VSMCs were not highlighted, the authors have kindly deposited the corresponding data for the four VSMCs clusters at https://github.com/LI-Yan-Ming/Circulation.-2020-142-1374-1388. Their analysis shows that the upregulation or downregulation of several of these markers is cluster-specific, with some clusters showing increased and others showing decreased expression of *ACTA2* (coding for αSMA) and *TAGLN* (coding for SM22 α) between control and TAA samples [497].

### 7.2. Mechanical and Structural Heterogeneity

Regional differences along the length of the aorta have been observed in regard to ECM composition, mechanical properties, and mechanical stresses [71,384,498]. For example, increased elastin content in the thoracic aorta relative to the abdominal aorta is necessary to support an increased need for the storage and release of elastic energy during systole and diastole to promote blood flow [71,384,499,500,501,502]. The distribution of circumferential and longitudinal mechanical stresses is also not uniform across the vessel; in particular, the proximal aorta is subjected to increased longitudinal strain because with each heartbeat, this region is both distended by blood exiting the heart and stretched across its length by the contraction of the left ventricle [503]. Studies of mouse models of TAA associated with fibrillin-1 and fibulin-4 deficiency show that aneurysmal dilatation associates with localized reduction in the ability to store elastic energy and an increased circumferential stiffness [384,387]. However, these parameters vary longitudinally and around the circumference of the vessel (inner vs. outer curvature) [504].

Additional variation exists transmurally across the thickness of the vessel. Elastin fibers tend to organize circumferentially in the middle portion of the media while assuming a more longitudinal distribution in regions closer to the adventitia; they also manifest a “waviness gradient”, with inner layers of elastic lamellae being “wavier” than outer layers, which is a feature necessary to compensate for the extra circumferential stretch born by the inner surface of the vessel [505,506]. The transmural distribution of proteoglycan is also not homogenous, with the concentrations being the highest toward the intimal and medial layer; this distribution has been proposed to regulate residual stresses in the arterial wall [43]. Moreover, the convection of soluble agents across the aortic wall is the result of hydraulic conductance that proceeds from the lumen toward the adventitia, with localized variations in concentrations that can be affected by a number of factors including blood pressure, wall permeability, and binding to local wall components (reviewed in reference [507]).

The interaction between mechanical heterogeneity combined with intrinsic differences in VSMCs (and perhaps adjacent endothelial cells) of a given lineage might contribute to rendering specific regions of the vessel, such as the proximal aorta, more susceptible to disease even when mutant gene products are expressed uniformly along the vessel. For example, SHF-derived VSMCs, which reside closest to the aortic root and are subject to the greatest longitudinal strain from the heartbeat, might rely more heavily on the activity of regulatory pathways that physiologically suppress the expression of mechanosensitive receptors such as AT_1_R (i.e., TGF-β signaling [411]). These regional differences need to be considered in the analysis of the molecular and cellular consequences of a given genetic variant, in the identification of therapeutic targets, and in development of targeted pharmacological therapies [488].

## 8. Summary and Conclusions

Integrity of the aorta is maintained by a complex and dynamic network of mechanical and biochemical signals. Genetic variants that cause hereditary aneurysm disorders affect the ability of VSMCs to receive and interpret these signals, resulting in phenotypic modulation in these cells and maladaptive remodeling of the ECM, eventually leading to mechanical failure of the vessel wall. Delineation of the molecular functions affected by TAA-associated mutations has identified molecular deficiencies that initiate the pathogenic process. These include defects in components of the elastin-contractile unit and positive effectors of canonical TGF-β signaling. The mechanisms by which these primary deficiencies interact with other pathways regulating VSMC homeostasis to produce both adaptive and maladaptive outcomes is the subject of current investigation. Although current therapeutic strategies focus on reducing mechanical stress, the identification of adverse signaling events that participate in maladaptive responses to the primary genetic insult may offer new therapeutic opportunities. Current experimental evidence suggests that compensatory signaling responses, such as those downstream of AT_1_R and TGF-β receptors, can have both adaptive and maladaptive consequences, depending on the cell types and phase of disease. Future areas of investigation include (a) human genetics efforts for the identification of new causative variants in genes not currently associated with TAA, which might offer fresh insights on the molecular defects that initiate aneurysm pathogenesis; (b) investigations of transcriptional and epigenetic changes at the single-cell level for each stage of disease using human samples, mouse models, and iPS-derived cell models, which may reveal the heterogenous and dynamic nature of responses to the primary genetic insult; and (c) the development of therapeutic strategies that target specific cellular subsets at specific phases of aortic remodeling and specifically promote the beneficial effects of compensatory responses while minimizing associated maladaptive consequences.

## Figures and Tables

**Table 1:** Genes classified as “definitive” in determining predisposition to heritable thoracic aortic disease based on the Clinical Genome Resource (ClinGen) framework [5,187] are listed in bold.

Genes Coding for Components of the Extracellular Matrix				
*Gene*	Inheritance	Protein Name	Primary Effect of TAA-Causing Variants	Disease (Phenotype MIM Number)
***FBN1*** [188]	AD	Fibrillin-1	Fibrillin-1 is an extracellular matrix glycoprotein that serves as the structural component of microfibrils. TAA-causing variants are predicted to impair protein synthesis, secretion, or incorporation of mutant fibrillin in the microfibrillar architecture.	Marfan Syndrome (154700)
*EFEMP2* [189,190]	AR	EGF-containing fibulin-like extracellular matrix protein 2 (Fibulin-4)	Fibulin-4 is necessary for elastic fiber formation. The known variants are predicted to result in defective maturation of elastic fibers in consequence of reduced cross-linking.	Cutis laxa type 1B (614437)
***COL3A1*** [191,192,193,194]	AD	Collagen α-1(III) chain	The alpha1 chains of type III collagen are a component of a fibrillar collagen that is found in the vascular system, often in association with type I collagen. The known variants are predicted to result in defective assembly. These mutations tend to cause dissection without preceding dilatation.	Vascular Ehlers–Danlos Syndrome (130050)
*BGN* [195]	X-linked	Biglycan	Biglycan is a secreted proteoglycan that interacts with other components of the ECM, including collagen type I/II/III/VI, elastin, microfibrils, and TGF-β. Although it is known that TAA-causing variants disrupt protein function, the exact effect on ECM deposition and/or TGF-β activity is not clear.	Meester–Loeys syndrome (300989)
*MFAP5* [196]	AD	Microfibrillar-associated protein 5	Microfibrillar-associated protein 5 is a component of microfibrils. The known variants are predicted to disrupt protein function, although the exact effect on ECM deposition or activity is not clear.	--
***LOX*** [197,198]	AD	Lysyl oxidase	Lysyl oxidase is an enzyme required for cross-linking and maturation of collagen and elastic fibers. TAA-causing variants are predicted to result in reduced enzymatic activity and thus reduced cross-linking.	Familial thoracic aortic aneurysm 10 (617168)
*ELN* [199]	AD	Tropoelastin	Tropoelastin self-assembles into polymer networks of elastin via a process that includes fibulin-4/5 complexes, lysyl oxidase, and other matrix proteins. It is the core component of elastic lamellae. A 25-bp deletion in exon 30 of *ELN* was found to be associated with aortic dilatation and rupture in some patients [199].	Cutis laxa (123700)
*FBN2* [200,201]	AD	Fibrillin-2	Fibrillin-2 is a component of microfibrils and may be involved in elastic fiber assembly. Most loss-of-function mutations cause congenital contractural arachnodactyly; evidence for a causal role in TAA is limited.	Congenital contractural arachnodactyly (121050)
**Genes coding for proteins involved in transduction of biochemical signals**				
Gene	Inheritance	Protein name	Primary effect of TAA-causing variants	Disease (PhenotypeMIM number)
***TGFBR1*** [202]	AD	TGF-β receptor type I	TGF-β receptor type I is one of the two components of the TGF-β Receptor heterodimer. Upon binding to TGF-β, it dimerizes with, and is phosphorylated by TGF-β Receptor II. Thus activated, it phosphorylates and activates SMAD2 and SMAD3 proteins. TAA-causing mutations are predicted to result in decreased kinase activity and thus reduced levels of SMAD phosphorylation.	Loeys–Dietz syndrome type 1 (609192)
***TGFBR2*** [202]	AD	TGF-β receptor type II	TGF-β receptor type II is one of the two components of the TGF-β receptor heterodimer. Upon binding to TGF-β, it phosphorylates and activates TGF-β receptor I, which in turns phosphorylates and activates SMAD proteins. TAA-causing mutations are predicted to result in decreased kinase activity and thus reduced levels of SMAD phosphorylation.	Loeys–Dietz syndrome type 2 (610168)
***SMAD3*** [203]	AD	Mothers against decapentaplegic drosophila homolog 3 (SMAD3)	SMAD3 is one of the major signal transduction molecules activated by TGF-β receptors through phosphorylation. TAA-causing mutations are predicted to result in either decreased protein levels or decreased SMAD3-dependent transcriptional activity.	Loeys–Dietz syndrome type 3 (613795)
*SMAD2* [204]	AD	Mothers against decapentaplegic drosophila homolog 2 (SMAD2)	SMAD2 is one of the major signal transduction molecules activated by TGF-β receptors though phosphorylation. TAA-causing mutations are predicted to result in decreased protein levels or decreased SMAD2-dependent transcriptional activity.	Loeys–Dietz syndrome type 6
***TGFB2*** [205,206]	AD	Transforming growth factor β-2 proprotein(TGF-β2)	TGF-β2 is one of the three TGF-β ligands that bind and activate signaling by TGF-β receptors. TAA-causing mutations are predicted to result in decreased protein levels or decreased binding to TGF-β receptors.	Loeys–Dietz syndrome type 4 (614816)
*TGFB3* [207]	AD	Transforming growth factor β-3 proprotein(TGF-β3)	TGF-β3 is one of the three TGF-β ligands that bind and activate signaling by TGF-β receptors. TAA-causing mutations are predicted to result in decreased protein levels or decreased binding to TGF-β receptors.	Loeys–Dietz syndrome type 5 (615582)
*SMAD4* [208,209]	AD	Mothers against decapentaplegic drosophila homolog 4 (SMAD4)	SMAD4 binds to phosphorylated SMAD2 and SMAD3 to facilitate translocation to the nucleus and transcriptional regulation downstream of TGF-β receptors. Variants associated with TAA are predicted to reduce SMAD4 activity and thus decrease TGF-β signaling output.	Juvenile polyposis/hereditary hemorrhagic telangiectasia syndrome (175050)
*LTBP3* [210]	AR	Latent transforming growth factor β binding protein 3 (LTBP-3)	LTBP-3 belongs to a family of proteins that regulate TGF-β activity by enabling its secretion and incorporation of its latent form into the ECM. They also participate in its conversion from latent to active form. Homozygous loss-of-function mutations associated with TAA are predicted to both decrease LTBP-3 levels in fibrillin-1–containing microfibrils and also decrease the overall secretion of TGF-β.	Dental anomalies and short stature syndrome (601216)
*SKI* [211,212]	AD	SKI protooncogene	SKI encodes a transcriptional repressor of TGF-β signaling. Mutations associated with TAA are predicted to disrupt the binding of SKI to SMAD proteins and other transcriptional co-regulators, resulting in a loss of inhibitory activity on the TGF-β signaling pathway.	Shprintzen–Goldberg syndrome (182212)
*NOTCH1* [213,214,215,216]	AD	Neurogenic locus notch homolog protein 1 (Notch 1)	Notch1 is one of the four receptors that are activated by binding to one of the membrane-bound Notch ligands (Delta-like 1, 3, and 4, and Jagged 1 and 2). In humans, loss-of-function mutations in Notch1 cause aortic valve disease and, in some limited cases, TAA.	Aortic valve disease (with or without thoracic aortic aneurysm) (109730)
**Genes coding for proteins involved in transduction of mechanical signals**				
Gene	Inheritance	Protein name	Primary effect of TAA-causing variants	Disease (PhenotypeMIM number)
***ACTA2*** [217]	AD	Smooth muscle actin α 2 (α-SMA)	α-SMA is a smooth muscle-specific form of actin that, as other actins, exists in two states: the globular monomeric G-actin and the structural filament F-actin. It is a major constituent of the cell contractile apparatus. TAA-associated mutations are predicted to result in structurally altered actin monomers and less stable actin filaments.	Familial thoracic aortic aneurysm 6 (611788)
***MYH11*** [62,218]	AD	Myosin-11 or Smooth muscle myosin heavy chain (SM-MHC)	SM-MHC is a subunit of the hexameric myosin protein complex, which consists of two heavy chain subunits and two pairs of non-identical light chain subunits. It functions as a major contractile protein, converting chemical energy into mechanical energy through the hydrolysis of ATP. TAA-associated mutations are predicted to impair the ability of the mutant myosin to polymerize into thick filaments and form a quaternary structure. Haploinsufficiency for *MYH11* does not appear to cause aneurysm.	Familial thoracic aortic aneurysm 4 (132900)
***MYLK*** [219]	AD	Myosin light chain kinase (MLCK)	MLCK is a calcium/calmodulin-dependent kinase that phosphorylates myosin regulatory light chains to facilitate myosin interaction with actin filaments and thus produce contractile activity. TAA-associated mutations are predicted to either cause haploinsufficiency or impair kinase activity.	Familial thoracic aortic aneurysm 7 (613780)
***PRKG1*** [220]	AD	cGMP-dependent protein kinase 1 (PKG-1)	PKG-1 is a cGMP-activated kinase that promotes the relaxation of VSMCs. It activates the phosphatase that dephosphorylates myosin regulatory light chains. One recurring gain-of-function variant in the *PRKG1* gene results in the constitutive activation and TAA.	Familial thoracic aortic aneurysm 8 (615436)
*FLNA* [221,222]	X-linked	Filamin-A	Filamin-A is an actin-binding protein that links membrane glycoproteins, including integrins, to actin filaments. It also serves as a scaffold and integrator for a wide range of cytoplasmic signaling proteins [223]. Loss-of-function mutations are associated with a broad range of congenital malformations and with increased risk of TAA [224].	Periventricular nodular heterotopia type 1 (300049)
*ARIH1* [225]	AD	E3 ubiquitin–protein ligase ARIH1 (Ari-1)	The Ari-1 protein is an E3-ubiquitin ligase that controls the degradation of SUN2, which is a component of the LINC (Linker of Nucleoskeleton and Cytoskeleton) complex. The LINC complex is involved in the coupling of mechanical signals to nuclear regulation, including chromatin and transcriptional regulation [111]. The ARI-1 variants associated with aneurysm are predicted to interfere with LINC complex function, although the mechanism remains unclear.	
**Unclear function or mechanism**				
Gene	Inheritance	Protein name	Primary effect of TAA-causing variants	Disease (phenotypeMIM number)
*SLC2A10* [226]	AR	Solute carrier family 2, facilitated glucose transporter member 10 (GLUT10)	GLUT10 is a member of the class III facilitative glucose transporter family. Loss-of-function mutations in this transporter have been associated with aneurysms of large and medium-sized arteries. The pathogenic mechanisms remain unclear, although effects on mitochondrial function, TGF-β signaling, and synthesis of ECM glycoproteins have been proposed [226,227,228].	Arterial tortuosity syndrome (208050)
*HCN4* [229,230]	AD	Potassium/sodium hyperpolarization-activated cyclic nucleotide-gated channel 4 (HCN4)	HCN4 is a member of the hyperpolarization-activated cyclic nucleotide-gated potassium channels. Loss-of-function mutations in this gene have been linked to sick sinus syndrome 2, which is characterized by atrial fibrillation with bradyarrhythmia as well as increased risk of aneurysm. The pathogenic mechanism remains unclear.	Sick Sinus Syndrome 2 (163800)
*MAT2A* [231]	AD	S-adenosylmethionine synthase isoform type-2 (METK2), also known as methionine adenosyltransferase II α (MAT-IIα)	MAT-IIα catalyzes synthesis of S-adenosylmethionine from methionine and ATP. Mutations that predispose individuals to TAA are predicted to reduce or disrupt the activity of the enzyme. It remains unclear how reduced levels of S-adenosylmethionine cause disease.	
*FOXE3* [232]	AD	Forkhead box protein E3 (FOXE3)	FOXE3 is a transcription factor of the forkhead family of transcription factors. Mutations associated with TAA are predicted to disrupt the forkhead domain and result in defective transcriptional regulation.	Aortic aneurysm, familial thoracic 11 (617349)

## Data Availability

Not applicable.
